# Postprandial glycaemia-lowering effect of a green tea cultivar Sunrouge and cultivar-specific metabolic profiling for determining bioactivity-related ingredients

**DOI:** 10.1038/s41598-018-34316-8

**Published:** 2018-10-30

**Authors:** Masafumi Wasai, Yoshinori Fujimura, Haruna Nonaka, Ryo Kitamura, Motoki Murata, Hirofumi Tachibana

**Affiliations:** 1 Research Laboratory, Nippon Paper Industries Co., Ltd, Tokyo, Japan; 2 Division of Applied Biological Chemistry, Department of Bioscience and Biotechnology, Faculty of Agriculture, Kyushu University, Fukuoka, Japan

## Abstract

Although the major green tea catechins can inhibit the activity of carbohydrate-hydrolyzing enzymes, there is a paucity of information describing the potential of other green tea ingredients and numerous green tea cultivars. Herein, we reveled that a green tea cultivar Sunrouge significantly suppressed the postprandial blood glucose level in mice. Unlike the most representative Japanese green tea cultivar, Yabukita, the suppression by Sunrouge was observed clearly during the initial period after oral dosing of starch. Sunrouge also strongly inhibited the carbohydrate-hydrolyzing enzymes α-glucosidase and α-amylase when compared with that of Yabukita and many other cultivars. Liquid chromatography–mass spectrometry (LC–MS)-based metabolic profiling (MP) of 42 Japanese green tea cultivars was performed. Multivariate statistical analysis enabled visualization of the differences among cultivars with respect to their ability to inhibit carbohydrate-hydrolyzing activities. Analysis of metabolites, contributing to the discrimination and prediction of the bioactivity of cultivars, showed that *O*-methylated catechins, epicatechin-3-*O*-(3-*O*-methyl) gallate (ECG3”Me) and epigallocatechin-3-*O*-(3-*O*-methyl) gallate (EGCG3”Me), were newly identified α-glucosidase inhibitors. Such ability was also observed in epigallocatechin-3-*O*-gallate (EGCG), epicatechin-3-*O*-gallate (ECG), delphinidin-3-*O*-glucoside and myricetin-3-*O*-glucoside. The amounts of these compounds in Sunrouge were higher than that in many other cultivars. These results suggest that Sunrouge has high potential for suppressing the elevation of the postprandial blood glucose level, and an MP approach may become a valuable strategy for evaluating the anti-hyperglycemic activity of green tea and for screening its active ingredients.

## Introduction

Diabetes is currently one of the most significant public health challenges worldwide. The number of people with diabetes is projected to rise to 439 million by 2030^1^. Nearly 90% of the cases of incident type 2 diabetes are a chronic metabolic disorder that is characterized by insulin resistance and high blood glucose levels^2^. Previous research suggested that food-derived phenolic compounds could delay glucose absorption by inhibition of carbohydrate-hydrolyzing enzymes, which would reduce the postprandial blood glucose level. This could be a suitable approach to treat and prevent type 2 diabetes^3^.

The major phenoic compounds of green tea (*Camellia sinensis* L.) are flavonoids, and more specifically catechins. Green tea catechins have been reported to reduce postprandial blood glucose levels after administration of starch in mice^4,5^. In addition, retrospective cohort study in Japan found that consumption of the green tea was associated with a reduced risk of type 2 diabetes^6^. The green tea cultivar Sunrouge is rich in anthocyanins and includes similar levels of catechins as the cultivar Yabukita, which comprises 76% of the tea growing area and is the most popular cultivar consumed in Japan^7,8^. Interestingly, Sunrouge has unique bioactivity and composition pattern when compared with that of other green tea cultivars^9,10^. However, there is a paucity of information about the postprandial glycaemia-lowering effects of Sunrouge and its active ingredients.

Mass spectrometry (MS) technique is widely used for metabolomics research and liquid chromatography–mass spectroscopy (LC–MS) can detect a wide range of low-molecular-weight compounds such as secondary metabolites^11^. Metabolic profiling (MP) can pinpoint the association between the metabolites and phenotype^12^. Coupled with chemometric methods, including principal component analysis (PCA) and orthogonal partial least-squares (OPLS) regression analysis, MP is often used for evaluating the nutritional value in plant cultivars and to identify compounds conferring beneficial properties^13^. This approach may also be useful for the unbiased evaluation of the pharmaceutical properties of crude plant extracts and to identify specific bioactive compounds in extracts. However, there is no report about MP-driven bioactivity evaluation of green tea extracts related to the postprandial glycaemia-lowering effect.

In this study, we investigated the feeding effect of the Japanese green tea cultivar, Sunrouge, on the postprandial blood glucose level in mice and attempted to screen the active ingredients to identify an inhibitor of carbohydrate-hydrolyzing enzymes from Sunrouge by cultivar-specific MP-based data-mining technique.

## Results

### Effect of Sunrouge and Yabukita extract powders on postprandial blood glucose in mice

**Figure 1:**
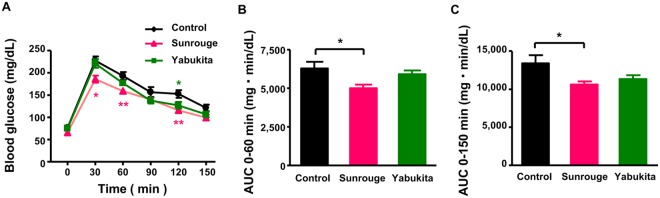
Effect of Sunrouge and Yabukita on postprandial blood glucose in mice. Mice were treated with Sunrouge extract powder or Yabukita extract powder (500 mg/kg b.w.) in combination with soluble starch (2,000 mg/kg b.w.). (**A**) Postprandial blood glucose levels are shown at each time point after oral dosing of starch. Calculated area under the curve (AUC) is shown during the initial time period (0–60 min) (**B**) and the whole time period (0–150 min) (**C**). Turkey’s Multiple Comparison Test, *P < 0.05, **P < 0.01 vs. Control.

Mice were orally administered with soluble starch (2,000 mg/kg b.w.) alone (Control), in combination with the extract powder (500 mg/kg b.w) of Sunrouge (anthocyanin-rich green tea cultivar), or in combination with the extract powder (500 mg/kg b.w) of Yabukita (most representative Japanese green tea cultivar). The administration of Sunrouge significantly suppressed the postprandial blood glucose level at the time points of 30, 60 and 120 min when compared with the control (Fig. 1A). In the Yabukita group, such a significant suppression was only observed at the 120 min time point. In AUC data, the significant suppression of the postprandial blood glucose level was observed in Sunrouge during both the initial time period (0–60 min; Fig. 1B) and the whole time period (0–150 min; Fig. 1C) after oral dosing of starch, but not in Yabukita. These results showed the different abilities of the two cultivars to inhibit the elevation of the postprandial blood glucose level. The results showed unequivocally that the Sunrouge (500 mg/kg b.w) was superior in inhibiting the postprandial blood glucose level when compared with the Yabukita cultivar.

### Inhibitory effect of green tea cultivars on carbohydrate-hydrolyzing enzymes

**Figure 2:**
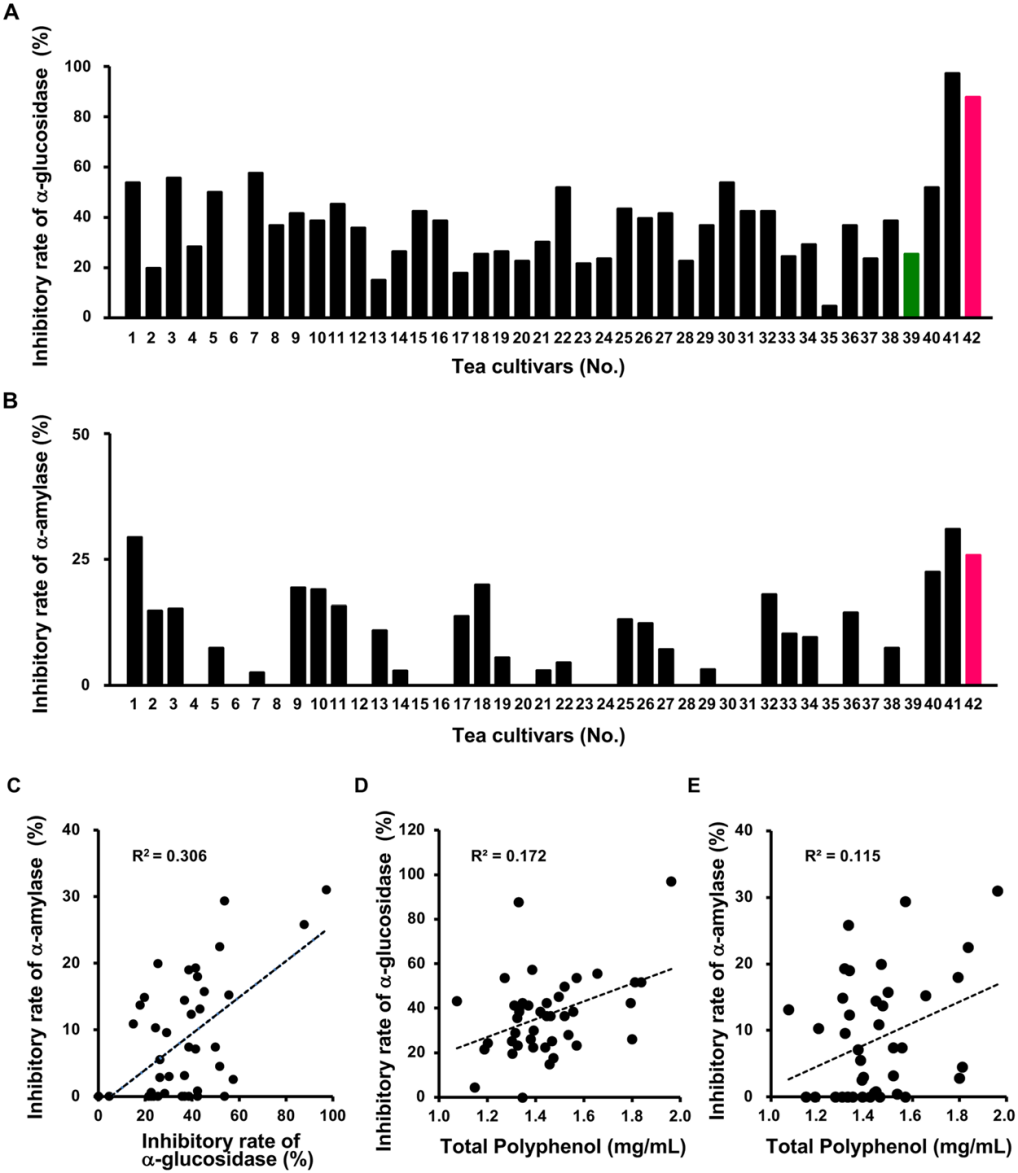
Effects of extracts from 42 kinds of green tea cultivars on glycolytic enzymes. Inhibitory effects of 42 green tea extracts on the activities of (**A**) α-glucosidase and (**B**) α-amylase in a cell-free system. (**C**) The correlation plot between the inhibitory rates of two glycolitic enzymes. The potential relationship between the total polyphenol content and the inhibitory rate of (**D**) α-glucosidase or (**E**) α-amylase of 42 cultivars.

Starch is metabolized by α-glucosidase and α-amylase, resulting in the formation of glucose. Inhibition of the activities of these enzymes may be involved in the suppressive effect of Sunrouge and Yabukita on the postprandial blood glucose level, and may account for the difference in their inhibitory activity. We then investigated the inhibitory effect of extracts from 42 Japanese green tea cultivars (Supplementary Table S1), including Sunrouge and Yabukita, on the activities of α-glucosidase and α-amylase in a cell-free system. Differences in the inhibitory activities of tested cultivars were observed for both α-glucosidase (Fig. 2A) and α-amylase (Fig. 2B). Cha Chuukanbohon Nou 6 (Nou 6, No. 41) and Sunrouge (No. 42) exhibited the strongest inhibitory activity of α-glucosidase among the cultivars, including Yabukita. Nou 6, Sunrouge, Seishin-oolong (No. 1) and Benifuuki (No. 40) exhibited the strongest inhibitory activity of α-amylase, whereas Yabukita showed no inhibitory activity. The number of non-active cultivars was different between both enzymes, with 1 cultivar inactive against α-glucosidase and 15 cultivars were inactive against α-amylase. Among the 42 cultivars, Nou 6 and Sunrouge showed strongest inhibitory activities toward both enzymes tested, but a clear correlation was not observed between both enzymes in a correlation plot analysis using all tea cultivars (Fig. 2C). Understanding of cultivar-specific properties, such as chemical composition and abundance, may reveal the reasons to why green tea cultivars, such as Nou 6 and Sunrouge, are capable of inhibiting the activity of glycolytic enzymes. Previously, studies on the relationship between polyphenols and glycolytic enzymes were reported^14^. Herein we evaluated the correlation between the total polyphenol contents of all green tea extracts and their α-glucosidase (Fig. 2D) or α-amylase (Fig. 2E) inhibitory rate. The correlation was not observed in both plot models, indicating that the inhibitory rates of green tea extracts on both α-glucosidase and α-amylase were not dependent on the amount of total polyphenols.

### LC–MS-based metabolic profiling to evaluate bioactivity of green tea cultivars and screen bioactivity-related ingredients

**Figure 3:**
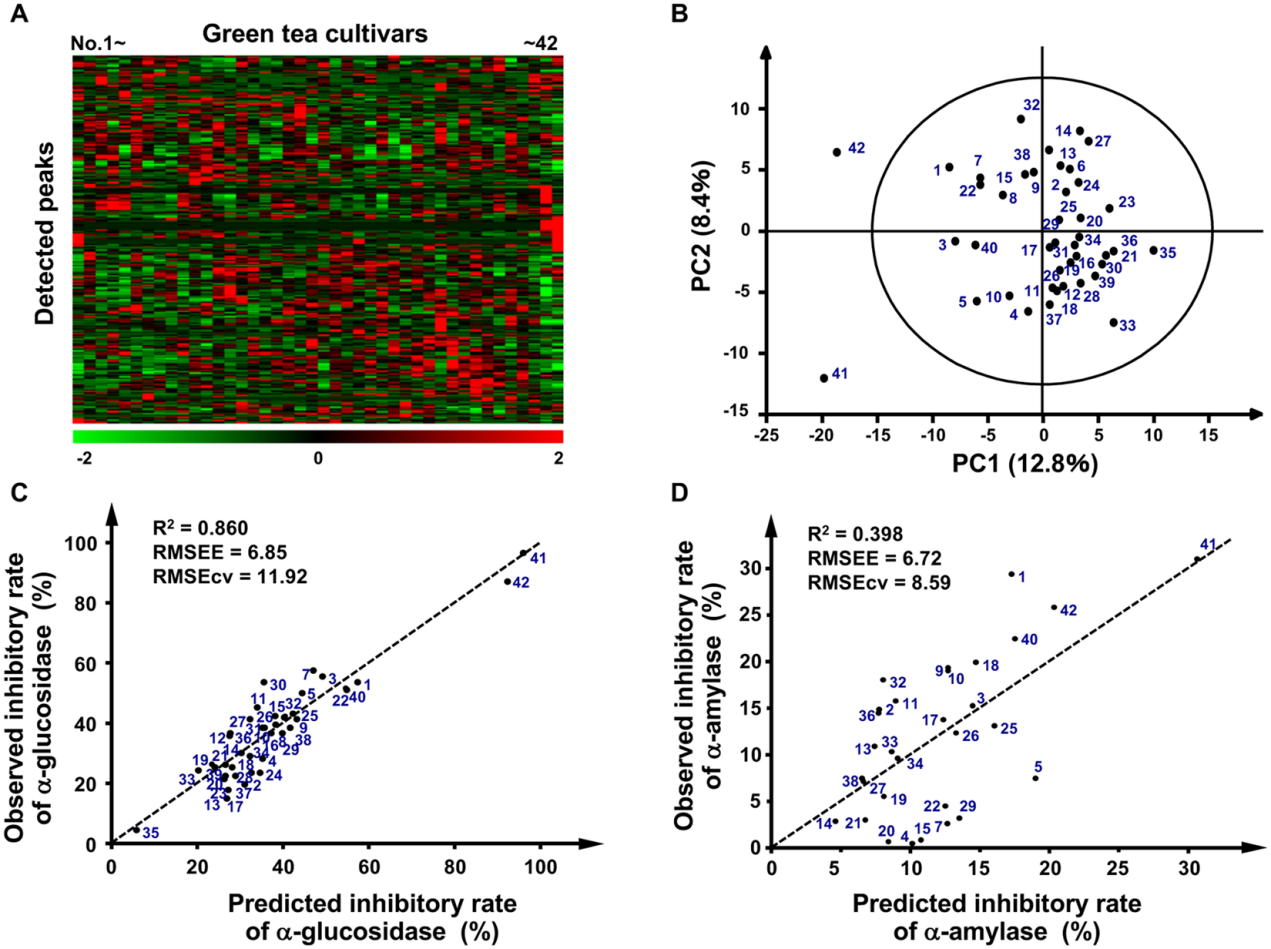
Multivariate statistical analysis of metabolic profiles derived from various tea extracts. (**A**) Heat map of 42 green tea cultivars. Columns represent the metabolic profile of single cultivars and rows represent 279 detected peaks in LC–MS measurement. (**B**) PCA score plot shows separate clustering of MS profiles corresponding to Nou 6 (No.41) and Sunrouge (No.42), and other cultivars. The bioactivity-prediction OPLS model was calculated from the LC–MS dataset of (**C**) 41 (α-glucosidase) or (**D**) 27 (α−amylase) tea samples, excluding cultivars that showed an inhibitory rate of 0%. *R*^2^, the goodness-of-fit parameter; RMSEE, the root mean squared error of the estimation; RMSEcv, the root mean squared error of cross validation.

To examine the potential relationship between the chemical composition of the extracts derived from 42 green tea cultivars and their bioactivity, these extracts were measured by LC–MS and subjected to multivariate statistical analysis. Metabolic profiles, consisting of 279 molecular features (Supplementary Table S2), differed markedly among green tea cultivars (Fig. 3A). The unsupervised and exploratory data analysis, the PCA score plot, showed that one comprised two cultivars, Nou 6 (No.41) and Sunrouge (No.42), with high carbohydrate-hydrolyzing enzyme-inhibiting potency, and another comprised the remaining cultivars, including Yabukita (No. 39) (Fig. 3B). These results suggest that chemical differences among cultivar extracts account for observed differences in bioactivity.

Although the PCA analysis provided an overview of all observations, the details of differences in each cultivar remained unclear. To determine whether bioactivity of the tea cultivars was correlated with their metabolic profiles, i.e., chemical composition, we created an OPLS regression model using composition profiles of green tea extracts and their bioactivity, such as the inhibitory activity of α-glucosidase or α-amylase. The regression model describing the inhibition of α-glucosidase activity using 41 cultivars (the non-active cultivar was excluded) showed a good correlation with the goodness-of-fit parameter *R*^2^ = 0.860, the root mean squared error of the estimation (RMSEE) = 6.85, and the root mean squared error of cross validation (RMSEcv) = 11.92 (Fig. 3C). In contrast, the regression model describing the inhibition of α-amylase activity using 27 cultivars showed a weak correlation with *R*^2^ = 0.398, RMSEE = 6.72 and RMSEcv = 8.59 (Fig. 3D). The error rate of α-glucosidase was 7.0% (RMSEE = 6.85/Maximum inhibitory rate = 97.17) and that of α-amylase was 21.7% (RMSEE = 6.72/Maximum inhibitory rate = 30.98). Composition profiles of green tea extracts correlated with both inhibitory activities, and the correlation of α-glucosidase was higher than that of α-amylase, indicating that the predictive performance of the inhibitory activity of α-glucosidase was higher than that of α-amylase in the OPLS regression model.

**Figure 4:**
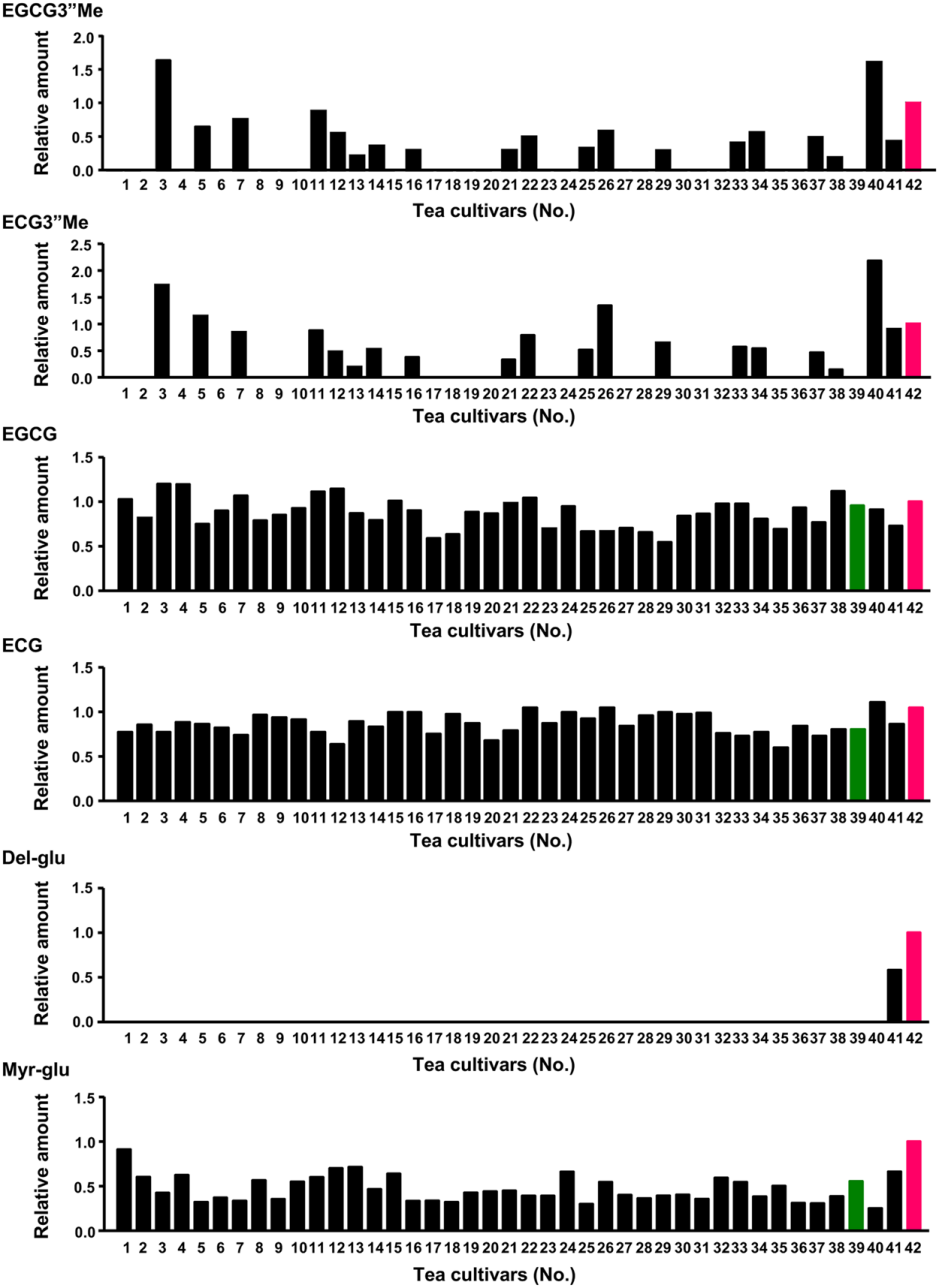
The relative amount of the identified compounds in 42 green tea extracts. Relative amounts were calculated from the intensity data of MS profiles of 42 green tea cultivars and represented as relative value of each cultivar to Sunrouge.

In the OPLS model, compounds that were highly relevant for explaining predicted inhibitory activity were also identified from VIP (variable importance in projection) values. Large VIP values correspond to the best explanations of predicted bioactivity. To screen candidates that contribute to the inhibitory activity, we selected compound peaks with high VIP values (>0.8) that their peak intensity was positively correlated with bioactivity of cultivars (Supplementary Table S2, peaks highlighted with red color). In this study, we did not focus the α-amylase model with poor predictive performance, but the α-glucosidase model with good performance, and 34 compound peaks were observed with high VIP values. Among them, six polyphenolic compounds, epigallocatechin-3-*O*-(3-*O*-methyl) gallate (EGCG3”Me), epicatechin-3-*O*-(3-*O*-methyl) gallate (ECG3”Me), epigallocatechin-3-*O*-gallate (EGCG), epicatechin-3-*O*-gallate (ECG), delphinidin-3-*O*-glucoside (Del-glu) and myricetin-3-*O*-glucoside (Myr-glu), were assigned using commercially available authentic standards (Supplementary Fig. S1). These six peaks were relatively abundant in the cultivar Sunrouge (No.42), which showed higher bioactivity (Fig. 4). In particular, the highest abundance of an anthocyanin Del-glu and a flavonol Myr-glu were found in Sunrouge. Although there were major green tea catechins, EGCG and ECG, in all cultivars, their *O*-methylated forms, EGCG3”Me and ECG3”Me, were detected in only 20 cultivars, including anthocyanin-rich cultivars, Sunrouge and Nou 6 (No. 41), and Benifuuki (No. 40) and its closely related species, Benihomare (No. 7), Benihikari (No. 5) and Benifuji (No. 3). In the control cultivar Yabukita (No.39) with poor bioactivity, these six peaks were zero or had lower abundance when compared with the corresponding results for Sunrouge. We also calculated correlation coefficients between the amounts of gallated catechins and the α-glucosidase inhibitory rates of 41 green tea cultivars by single regression model; ECG: 0.317, EGCG: 0.246, ECG3”Me: 0.502 and EGCG3”Me: 0.472 (Supplementary Table S2). Four catechins were positively correlated with the α-glucosidase inhibitory rate. The values of methylated catechins, ECG3”Me and EGCG3”Me, were higher than that of their non-methylated types, ECG and EGCG. On the other hand, non-gallated catechins, epicatechin (EC) and epigallocatechin (EGC) (Supplementary Fig. S2), were negatively correlated with the α-glucosidase inhibitory rate, and their correlation coefficients were −0.459 and −0.525, respectively (Supplementary Table S2). Thus, the contribution rate was dependent on the type of catechins. Intriguingly, the correlation coefficient of the total amount of four catechins (ECG + EGCG + ECG3”Me + EGCG3”Me) was 0.538 (Supplementary Fig. S3A) and this value was higher than that of each catechin. This data showed a statistical significance (*P* = 0.00028), indicating that the total amount of four gallated catechins was correlated with the inhibitory rate of α-glucosidase. Further analysis of bioactive composition based on the combination of multiple tea compounds may provide the accurate contribution rate of each compound in complex multicomponent systems such as green tea extracts.

### Effects of the selected compounds on α-glucosidase activity

To clarify whether the above-mentioned compounds can possess the inhibitory activity of glycolytic enzymes, we examined the inhibitory effect of six identified compounds, catechins (EGCG3”Me, ECG3”Me, EGCG and ECG) and glycoside analogues (Del-glu and Myr-glu), and their related two aglycones (delphinidin (Del) and myricetin (Myr)), on α-glucosidase activity in a cell-free system. Table 1 shows the half inhibitory concentration (IC_50_) values toward α-glucosidase. All compounds inhibited α-glucosidase, and the highest inhibitory potency was observed in EGCG3”Me and Myr. Although the inhibitory ability of Myr and Del was reduced by glucoside conjugation, inversely, another conjugation, *O*-methylation, enhanced the inhibitory ability of EGCG and ECG. An alteration rate of ECG, 4.2-fold enhancement (IC_50_: 61.1 μM/14.7 μM), was higher than that of EGCG, 1.6-fold enhancement (IC_50_: 13.3 μM/8.1 μM). In addition, we showed data on α-glucosidase inhibitory rates of EGCG, ECG, EGCG3”Me and ECG3”Me at the concentration of 10 μM (Supplementary Fig. S4). The order of the inhibitory rate was EGCG3”Me > EGCG > ECG3”Me > ECG. This order was consistent with data on their IC _50_ values (Table 1). Significant difference was observed among the four catechins. Most intriguingly, we found for the first time that ECG3”Me and EGCG3”Me acted as α-glucosidase inhibitors, which were present abundantly in the strongly bioactive cultivar Sunrouge, but present in zero levels in the poor-bioactive cultivar Yabukita. Taken together, these findings suggested that the inhibition of α-glucosidase activity by multiple polyphenols selected from the bioactivity-prediction OPLS regression model may be involved in the suppressive effect of Sunrouge on the postprandial blood glucose level, and the inhibitory potency against α-glucosidase may contribute partly to the difference in bioactivity *in vivo* between Sunrouge and Yabukita.

**Table 1:** IC_50_ of several identified compounds against α-glucosidase.

Compound	α-Glucosidase
	IC_50_ (μM)
Myr	4.4
Myr-glu	>100
Del	31.6
Del-glu	>100
EGCG	13.3
ECG	61.1
EGCG3”Me	8.1
ECG3”Me	14.7

## Discussion

Various kinds of green tea compounds, except for the most abundant ingredients of catechins, and numerous green tea cultivars are known, but it remains unclear whether these tea compounds and cultivars have hypoglycemic potency by inhibiting carbohydrate-hydrolyzing enzymes. Herein we have demonstrated for the first time that a metabolomics approach, cultivar-specific MP, can be used to evaluate the hypoglycemic-related activity of various Japanese green tea cultivars and to identify bioactive factors. A green tea cultivar Sunrouge (No. 42) was found to effectively suppress the postprandial blood glucose level after oral administration of starch in mice. This cultivar strongly inhibited the carbohydrate-hydrolyzing enzymes, α-glucosidase and α-amylase, when compared with the corresponding results for other green tea cultivars, including the control cultivar Yabukita (No. 39). A previous cohort study of Japanese found that consumption of green tea was associated with a reduced risk of type 2 diabetes^6^. Considering that Yabukita comprises 76% of the tea growing area in Japan, our results suggest that the consumption of Sunrouge tea may reduce the risk for type 2 diabetes more effectively than Yabukita. LC–MS-based MP facilitated the identification of several bioactive factors that were enriched in Sunrouge rather than Yabukita. These observations suggest that the strong inhibitory effect of Sunrouge may be due to the presence of α-glucosidase inhibitors such as EGCG, ECG, EGCG3”Me, ECG3”Me, Myr-glu and Del-glu. Although the inhibitory activity of individual compounds has been partially reported^15–18^, little is known about the inhibitory activity of green tea cultivar, including all aforementioned compounds. Interestingly, we succeeded for the first time in identifying ECG3”Me and EGCG3”Me as new α-glucosidase inhibitors. The inhibitory activities of Myr-glu and Del-glu were weak compared with that of the other compounds, but this study was the first attempt to examine their inhibitory potency against α-glucosidase. It is known that Myr-glu and Del-glu are hydrolyzed to aglycone in small intestine where α-glucosidase is located^19^. The α-glucosidase inhibitory activity of such aglycones was higher than that of their intact forms as shown here. Therefore, we can’t exclude the possibility that Myr-glu and Del-glu in Sunrouge may lead to the effective inhibition of α-glucosidase activity through their deglycosylated forms in small intestine. Among polyphenols tested here, myricetin showed the highest inhibitory rate (Table 1). This activity was higher than that of EGCG, the most abundant green tea polyphenols. We also performed the oral starch tolerance test in mice with myricetin or EGCG (Supplementary Fig S5). Both green tea-related polyphenols suppressed the elevation of postprandial blood glucose level. The same tendency of these *in vitro* evaluation of enzymatic activity and *in vivo* oral starch tolerance test was observed in the green tea cultivar Sunrouge. These results suggest that *in vitro* α-glucosidase inhibitors selected by OPLS regression analysis possess *in vivo* anti-hyperglycemic activity. We believe that proposed MP method, combination of *in vitro* evaluation and OPLS regression analysis, may become an effective strategy for predicting bioactive cultivars and selecting bioactive compounds. On the other hand, the present findings can’t demonstrate direct relationship between the *in vivo* anti-hyperglycemic activity and the *in vivo* carbohydrate-hydrolyzing activities. In the future, studies for elucidating such a relationship are required to improve the performance of MP technique, especially in the predictability of OPLS model and the accuracy of identification of multiple bioactive compounds and their effective combination.

In this study, we found that *O*-methylation enhanced α-glucosidase inhibition by EGCG and ECG. EGCG is known to have the highest inhibitory ability toward α-glucosidase among all major catechins, and is classified into the high inhibition group along with myricetin, quercetin, genistein and cyanidin among flavonoids^16^. Our data showed that *O*-methylated ECG, ECG3”Me, had approximately the same inhibitory effect as EGCG and that *O*-methylated EGCG, EGCG3”Me, displayed higher inhibitory activity than EGCG. The order of methylated catechins (EGCG3”Me and ECG3”Me) was Benifuuki > Sunrouge > Nou 6 > Yabukita (Fig. 4). In case of the total amounts of 6 positively correlated compounds (EGCG + ECG + EGCG3”Me + ECG3”Me + Del-glu + Myr-glu), the order was Benifuuki ≒ Sunrouge > Nou 6 > Yabukita (Supplementary Fig. S3B). This order was inconsistent with that of carbohydrate-hydrolyzing activity (Fig. 2). In the present study, we identified 4 compounds such as theanine, caffeine, EC and EGC (Supplementary Fig. S7), showing negative correlation with α-glucosidase inhibitory rate and having large VIP values (>0.8) selected in OPLS regression analysis (Supplementary Table S2). In fact, theanine, the highest correlated compound (*R* = −0.576), elevated the activity of α-glucosidase, and the inhibitory rate of Sunrouge was significantly attenuated upon treatment with theanine (Supplementary Fig. S2). In case of the total amount of 4 negatively correlated compounds (theanine + caffeine + EC + EGC), the order was Yabukita > Benifuuki > Sunrouge > Nou 6 (Supplementary Fig. S3C). In addition, this order was also observed in EC or EGC alone (Supplementary Fig. S7). These results were consistent with the order of the inhibitory rate of α-glucosidase. The amount of methylated catechins highly contributed to the bioactivity-predictive OPLS regression model was positively correlated with the inhibitory rate of α-glucosidase when all cultivars were subjected to correlation analysis. However, such compounds could not always explain the difference in bioactivity among limited numbers of cultivars such as Yabukita, Benifuuki, Nou 6 and Sunrouge. In contrast, combination of 4 negatively correlated compounds (theanine + caffeine + EC + EGC) with large VIP values (>0.8) was useful information for understanding the difference in bioactivity among four cultivars (No. 39–42).

In future, such information may be useful for the development of markers to produce new cultivars with greater bioactivity and screen for bioactive tea cultivars. At least, we can predict the potential bioactivity of numerous tea cultivars by analyzing their composition data with OPLS regression models without the requirement for additional bioactivity assays. The PCA score plot of MS profiles (Fig. 3B) showed that two unique cultivars, Sunrouge (No. 42) and Nou 6 (No. 41), were separated from a cluster consisting of the other green tea cultivars (*Camellia sinensis*), such as Yabukita. This result indicated that these two cultivars had unique chemical composition. An anthocyanin-rich cultivar Nou 6 was bred from seedling of *Camellia taliensis*, which has been reported to contain characteristic phenolic constituents^20^. Sunrouge was generated by natural cross-breeding of Nou 6 with *Camellia sinensis*^7,8^. Therefore, both the strong inhibitory activity of α-glucosidase and α-amylase (Fig. 2A,B), and the unique composition (Fig. 3A,B) of Sunrouge and Nou 6 might result from a hybrid variety of *C. sinensis* and *C. taliensis*. It was known that Benifuuki (No. 40) possessed various health-promoting effects and unlike Yabukita is an abundant cultivar of *O*-methylated catechins, which are mostly responsible for its diverse bioactivities^21–24^. As shown in Fig. 4, higher amounts of *O*-methylated catechis were observed in this cultivar and its closely related species (No. 3, 5, 7), which showed a higher inhibitory rate of α-glucosidase (Fig. 2A,B). Furthermore, an oolong tea-suitable type cultivar, Ohba-oolong (No. 11), also showed relatively high abundance of *O*-methylated catechis and relatively high bioactivity. In contrast, another cultivar, Seishin-oolong (No. 1) had higher inhibitory rates toward both enzymes, but *O*-methylated catechis were not detected. This result suggests that another identified compound, Myr-glu enriched in this cultivar and Sunrouge, and other unidentified compounds may be involved in the inhibitory activity of Seishin-oolong. These findings should be helpful for choosing cultivars with postprandial glycaemia-lowering effects and glycolytic enzyme inhibitory activity. Further analysis of cultivar-specific composition profiles and bioactivity-related compounds, especially identification of many non-assigned compound peaks with large VIP values selected in bioactivity-predictive OPLS regression models (Fig. 3), is required for elucidating more precise bioactivity-correlated compound patterns that will be useful for unraveling complex mechanisms of action of multicomponent green tea extracts and their application to functional foods, nutraceuticals and botanical drugs.

In OPLS regression analysis (Fig. 3C,D), the predictive performance of α-amylase was lower than that of α-glucosidase, indicating that the number and kinds of compound peaks correlated to the inhibitory activity of α-amylase were less than that of α-glucosidase. Further analysis of different peak profiles obtained by other LC–MS conditions will become an effective strategy for constructing more precise bioactivity-prediction models and the subsequent identification of more effective bioactive compounds. Recently, we have reported that the MP-based data-mining technique could be used for effectively identifying bioactive chemical combinations in green tea extracts^10,25^. An introduction of this chemical combination-oriented approach to the present study may open new avenues for investigating the potential relationship between the bioactivity of crude green tea extracts and their multiple coexisting ingredients, and for determining effective chemical combinations for prediction of glycolytic enzyme inhibitory activity.

Taken together, the present study suggests that a unique Japanese green tea cultivar Sunrouge significantly suppresses the elevation of the postprandial blood glucose level by inhibiting the activity of both α-glucosidase and α-amylase. Therefore, daily consumption of Sunrouge may be an effective approach to prevent type 2 diabetes because of its postprandial glycaemia-lowering effect. In addition, our results may provide a new method, the cultivar-specific MP technique, capable of easily screening bioactivity-related compounds in crude green tea extracts without additional fractionation or purification.

## Methods

### Preparation of tea extract powders for starch tolerance test

Sunrouge leaves were purchased from Tokunoshimaseicha Co., Ltd (Kagoshima, Japan) and Yabukita leaves were purchased from Issin LLC (Shizuoka, Japan). Sunrouge and Yabukita extract powders were produced from Tokiwa Phytochemical Co. Ltd (Chiba, Japan). The catechin content of Sunrouge and Yabukita extract powders was analyzed by the Japan Food Research Laboratories (Supplementary Table S2). Sunrouge and Yabukita extract powders contained catechins at 230.8 and 201.6 mg/g, respectively.

### Preparation of tea samples for metabolic profiling analysis

Forty-two kinds of the major Japanese green tea cultivars (Supplementary Table S1) were provided by the Institute of Fruit Tree and Tea Science, NARO, Japan. Dried leaf powder of each cultivar (200 mg) was added to 10 mL boiling water for 10 min. The extract was centrifuged at 1,680 × *g* for 10 min. The supernatant was filtered using a 0.22-μm filter (Sartorius Stedim Biotech, Göttingen, Germany).

### Chemicals

Epigallocatechin-3-*O*-gallate (EGCG), epicatechin-3-*O*-gallate (ECG), epicatechin (EC), epigallocatechin (EGC), L-theanine, caffeine and myricetin (Myr) were purchased from Sigma-Aldrich (St. Louis, MO, USA). Delphinidin (Del) was purchased from Extrasynthese (Genay, France). Epicatechin-3-*O*-(3-*O*-methyl) gallate (ECG3”Me) was purchased from Nagara Science (Gifu, Japan). Delphinidin-3-*O*-glucoside (Del-gul) was purchased from Tokiwa Phytochemical (Chiba, Japan). Myricetin-3-*O*-glucoside (Myr-glu) was kindly provided by Dr. Toshio Miyase (School of Pharmaceutical Sciences, University of Shizuoka). Epigallocatechin-3-*O*-(3-*O*-methyl) gallate (EGCG3”Me) was kindly provided by Dr. Hiroshi Tanaka (Department of Applied Chemistry, Graduate School of Science and Engineering, Tokyo Institute of Technology).

### Starch tolerance tests in mice

Nine-week-old male ICR mice were purchased from Kyudo Company (Saga, Japan). All mice were housed and maintained in a temperature and humidity controlled room with a 12-h light-dark cycle (light from 8 am to 8 pm). All animal studies were performed in accordance with the law (protocol no. 105) and notification (protocol no. 6) of the Japanese government for the welfare of experimental animals. The study protocol was approved by the Animal Care and Use Committee, Kyushu University (Kyushu University, Scientific Research Promotion Division). Mice were randomly divided into 3 groups (*n* = 8) with equivalent mean body weight and starved overnight. Mice were orally administered with soluble starch (2,000 mg/kg b.w.) (Wako, Osaka, Japan) alone, or in combination with a test sample. All solutions were made in distilled water. Blood was sampled from the tail vein at before (*t* = 0), 30, 60, 90, 120 and 150 min after administration. The blood glucose level was measured with the Medisafe Mini GR-102 (Terumo Co., Tokyo, Japan) and the areas under the curves (AUC) were calculated.

### Assay of α-glucosidase activity

α-Glucosidase from *Saccharomyces cerevisiae* (Sigma-Aldrich) was dissolved in phosphate buffered saline (PBS) at a concentration of 0.01 mg/mL. Tea extracts were diluted 1:10 with distilled water. Tea extracts or various concentrations of flavonoids in DMSO were mixed 1:1 with the α-glucosidase solution. The α-glucosidase activity of the mixture was measured by the QuantiChrom™ α-Glucosidase Assay Kit (Bio Assay Systems, Hayward, CA, USA) according to the manufacturer’s instructions.

### Assay of α-amylase activity

α-Amylase from porcine pancreas (Sigma-Aldrich) was dissolved in PBS at a concentration of 0.06 mg/mL. Tea extracts were mixed 15:100 with the assay buffer from the MaxDiscovery™ Amylase Assay Kit (Bioo Scientific, Texas, USA). The α-amylase activity of the mixture was measured according to the manufacturer’s instructions.

### Determination of total phenolic content

Total phenolic content was determined using a modified version of the Folin-Ciocalteu method^26^. Tea extracts were diluted 1:10 with distilled water. Samples (20 μμL) were mixed with 150 μL of Folin & Ciocalteu’s Phenol Reagent (Wako, Osaka, Japan) followed by 150 μL of 6% Na_2_CO_3_ solution. After incubation at room temperature for 90 min, the absorbance of the samples was measured at 725 nm using a microplate reader (BioTek Instruments, Inc., USA). Total phenolic content was calculated from a calibration curve using gallic acid (Nacalai Tesque, Kyoto, Japan) as the standard.

### LC–MS analysis

Green tea extracts were diluted 1:10 with distilled water and subjected to non-targeted LC–MS analysis with an IT-TOF instrument (Shimadzu, Kyoto, Japan). The equipment was fitted with a L-column 2 ODS (2.1 mm I.D. × 150 mm, 3 μm; CERI, Saitama, Japan) maintained at 40 °C. The mobile phase solvents were: solvent A, H_2_O (0.05% formic acid) and solvent B, acetonitrile (0.05% formic acid). The gradient program was as follows: 0–2 min (A:B 95:5, v/v), 2–3.5 min (80:20, v/v), 3.5–10 min (60:40, v/v), 10–13 min (0:100, v/v), 13–15.5 min (0:100 v/v), 15.5–16 min (95:5 v/v), 16–20 min (95:5 v/v). The flow rate was 0.15 mL/min and the injection volume was 5 μL. The MS parameters were: electrospray ionization source in positive and negative modes with survey scans at *m/z* 100–700, nebulizing gas flow rate of 1.5 L/min, drying gas pressure of 0.2 MPa, capillary voltage of + 4.5/–3.5 kV, CDL temperature of 200 °C and heat block temperature of 200 °C.

### Multivariate statistical analysis

For all LC–MS datasets, peak picking, alignment and deletion of isotopic peaks were processed using Profiling Solution software (Shimadzu, Kyoto, Japan). All data in either positive or negative ion mode were separately normalized to the total ion counts (TIC) of each sample. After integration of TIC-normalized variables, the normalized datasets were analyzed using SIMCA13 (Umetrics, Umea, Sweden) for multivariate statistical analysis. The data matrix used here is listed in Supplementary Table S2. To obtain an overview of the sample features such as similarity or dissimilarity in metabolic profiles among 42 green tea cultivars, an unsupervised method, principal component analysis (PCA), was performed. The supervised method, orthogonal partial least squares (OPLS) regression analysis, was also performed to examine the potential relationship between metabolite profiles of tea cultivars and their ability to inhibit carbohydrate-hydrolyzing activities such as α-glucosidase and α-amylase, and isolate the potential compounds to predict the inhibitory effect of green tea cultivars. A heat map was generated by the statistical package MultiExperiment Viewer (MeV v4.8) (http://www.tm4.org/mev/). This summarizes the Z-score values of 279 compound peaks, which shows differences in metabolite profiles among cultivars. Metabolite peaks were assigned by MS/MS analysis or by searching their accurate masses using online metabolite databases (KEGG: http://www.genome.jp/kegg/; METLIN: http://metlin.scripps.edu/; MassBank: http://www.massbank.jp/).

### Statistical analysis of bioassays

Data are presented as means ± standard errors of the means. All data were analyzed using Tukey’s test. Prism software (GraphPad, Inc., San Diego, CA, USA) was used for the analysis. A *P* level < 0.05 was considered significant.

## Electronic supplementary material

Supplemental Information

Supplementary Information Table S2
