# Supplementary material for: Postprandial glycaemia-lowering effect of a green tea cultivar Sunrouge and cultivar-specific metabolic profiling for determining bioactivity-related ingredients
Source: Sci Rep. 2018 Oct 30;8:16041. doi: 10.1038/s41598-018-34316-8 (PMC6207662; doi:10.1038/s41598-018-34316-8)
Supplement: Supplementary file 1 — Supplemental Information [file 41598_2018_34316_MOESM1_ESM.pdf]

Postprandial glycaemia-lowering effect of a green tea cultivar Sunrouge and cultivar-specific metabolic profiling for determining bioactivity-related ingredients

Masafumi Wasai[a,b], Yoshinori Fujimura[b], Haruna Nonaka[b], Ryo Kitamura[b], Motoki Murata[b], Hirofumi Tachibana[b,*]

[a]Research Laboratory, Nippon Paper Industries Co., Ltd, Japan

[b]Division of Applied Biological Chemistry, Department of Bioscience and Biotechnology, Faculty of Agriculture, Kyushu University, Fukuoka, Japan

[*]Corresponding author: Division of Applied Biological Chemistry, Department of Bioscience and Biotechnology, Faculty of Agriculture, Kyushu University, 744 Motooka, Nishi-ku, Fukuoka 819-0395, Japan. Phone & Fax: +81-92-802-4749; E-mail: tatibana@agr.kyushu-u.ac.jp

Running title: Anti-hyperglycemic green tea cultivar 'Sunrouge'

Table S1. Forty-two kinds of the representative Japanese green tea cultivars.

No.	Cultivar	No.	Cultivar
1	Seishin-oolong	22	Yamatomidori
2	Fukumidori	23	Asatsuyu
3	Benifuji	24	Toyoka
4	Minekaori	25	Yaeho
5	Benihikari	26	Ujihikari
6	Minamikaori	27	Ooiwase
7	Benihomare	28	Gokou
8	Izumi	29	Surugawase
9	Fuusyum	30	Samidori
10	Tamamidori	31	Komakage
11	Ohba-oolong	32	Hatsumomiji
12	Seishintaipan	33	Ryofuu
13	Kuritawase	34	Minamisayaka
14	Syunmei	35	Saemidori
15	Sayamamidori	36	Okuyutaka
16	Asagiri	37	Okumidori
17	Hokumei	38	Yutakamirodi
18	Asahi	39	Yabukita
19	Sayamakaori	40	Benifuuki
20	Meiryoku	41	Cha Chuukanbohon Nou 6
21	Kanayamidori	42	Sunrouge

Table S3. Composition of tea extract powders.

Ingredient	Sunrouge extract powder (mg/g)	Yabukita extract powder (mg/g)
Catechin	2.9	6.9
Catechin gallate	0.2	1.3
Epicatechin	16.0	21.0
Epicatechin gallate	21.0	7.4
Gallocatechin	4.7	30.0
Gallocatechin gallate	2.3	11.0
Epigallocatechin	59.0	82.0
Epigallocatechin gallate	120.0	42.0
Epigallocatechin-3-*O*-(3-*O*-methyl)gallate	4.7	0
Total catechins	230.8	201.6

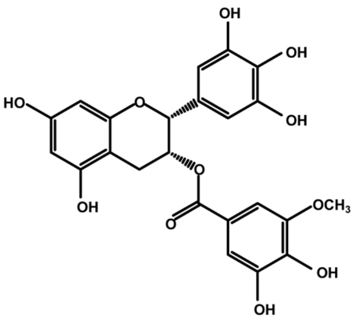

Epigallocatechin-3-*O*-(3-*O*-methyl) gallate (EGCG3"Me)

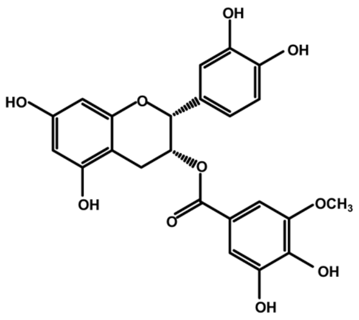

Epicatechin-3-*O*-(3-*O*-methyl) gallate (ECG3"Me)

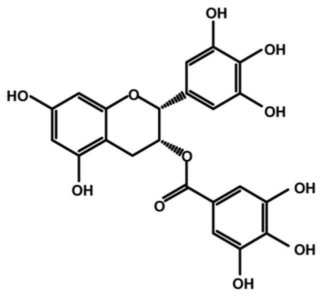

Epigallocatechin-3-*O*-gallate (EGCG)

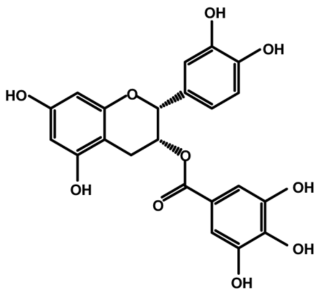

Epicatechin-3-*O*-gallate (ECG)

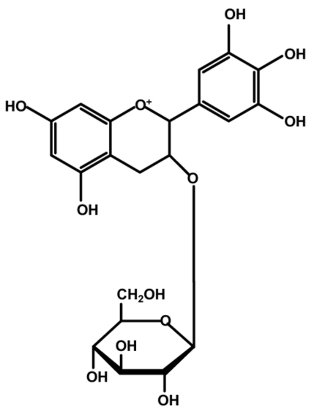

Delphinidin-3-*O*-glucoside (Del-glu)

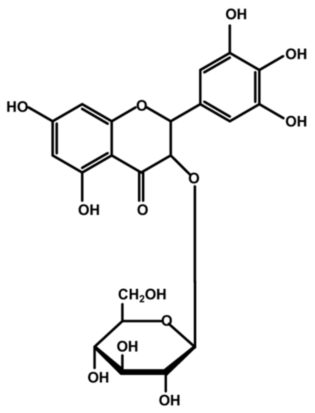

Myricetin-3-*O*-glucoside (Myr-glu)

Fig. S1. Chemical structures of compounds, showing positive correlation with α-glucosidase inhibitory rate, that contributed strongly to the construction of the bioactivity-prediction OPLS regression model.

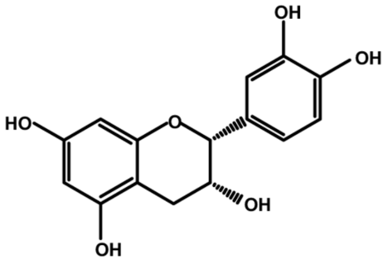

Epicatechin (EC)

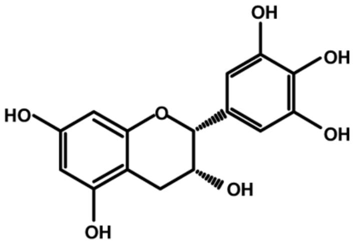

Epigallocatechin (EGC)

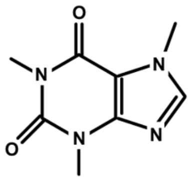

Caffeine

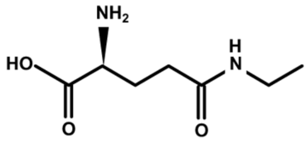

Theanine

Fig. S2. Chemical structures of compounds, showing negative correlation with α-glucosidase inhibitory rate, that contributed strongly to the construction of the bioactivity-prediction OPLS regression model.

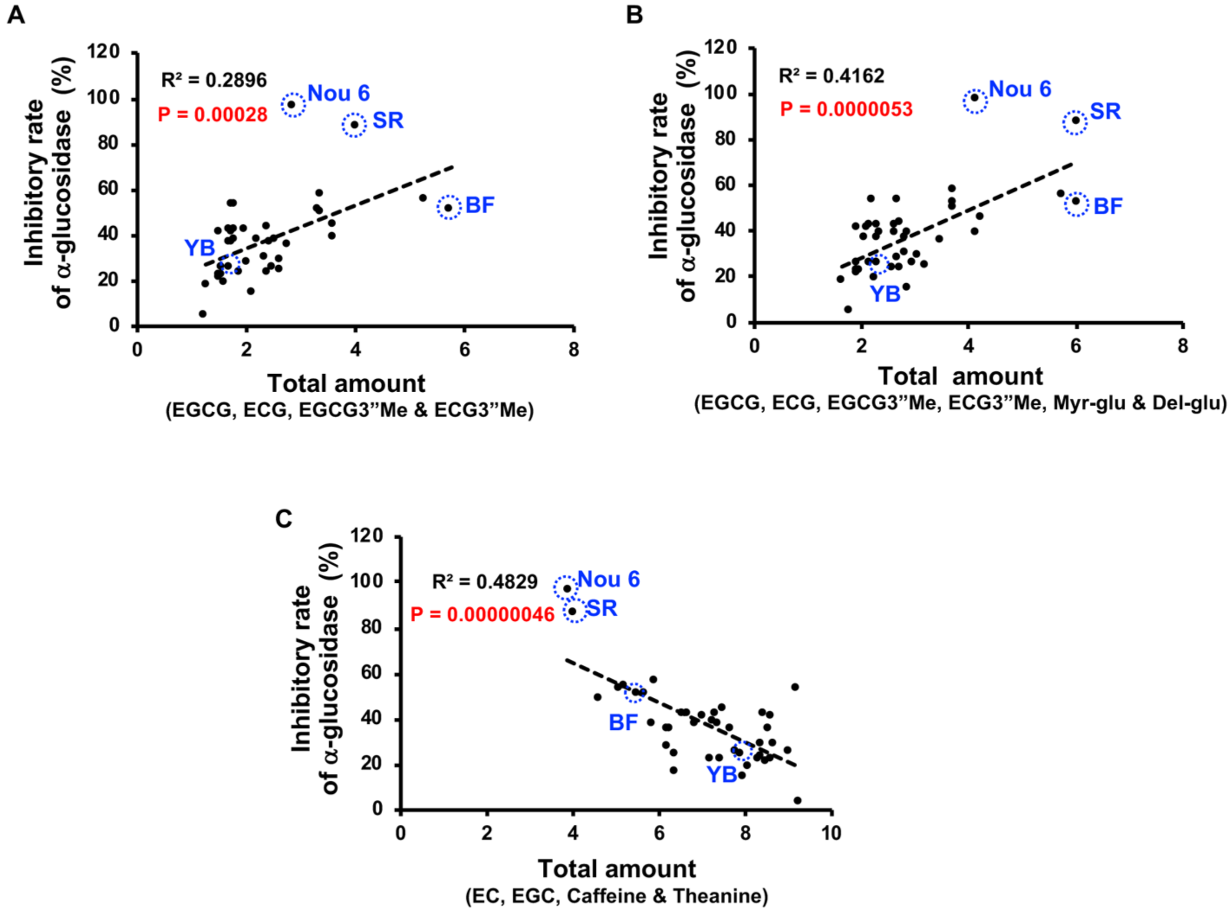

Fig. S3. The correlation plot between the total amounts of the representative compounds with large VIP values and the α-glucosidase inhibitory rates of 41 green tea cultivars. Single regression model was created to examine the potential relationship between the total amounts of 4 or 6 compounds (**A**: EGCG + ECG + EGCG3"Me + ECG3"Me; **B**: EGCG + ECG + EGCG3"Me + ECG3"Me + Myr-Glu + Del-glu; **C**: EC + EGC + Caffeine + Theanine) and the α-glucosidase inhibitory rates of 41 green tea cultivars. (**A** and **B**) Positivly correlated compounds with the α-glucosidase inhibitory rates. (**C**) Negatively correlated compounds. SR: Sunrouge; Nou 6: Cha Chuukanbohon Nou 6; BF: Benifuuki; YB: Yabukita.

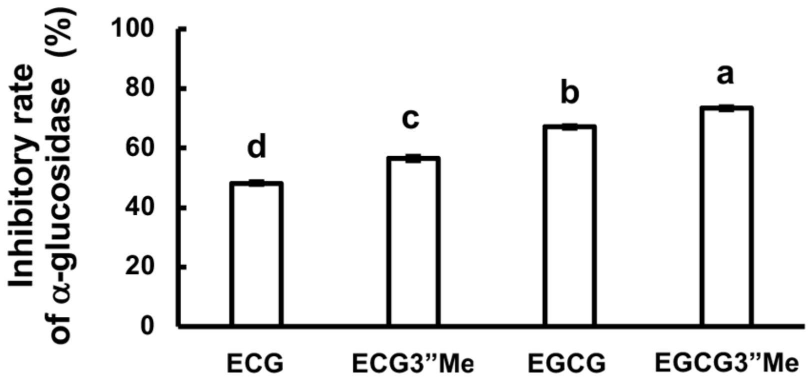

Fig. S4. The effect of gallated catechins on α-glucosidase activity. The activity of α-glucosidase was measured using each gallated catechin (ECG, ECG3"Me, EGCG or EGCG3"Me) at the concentration of 10 μM. Turkey's Multiple Comparison Test, n=3. Different letters are statistically different, $P < 0.05$.

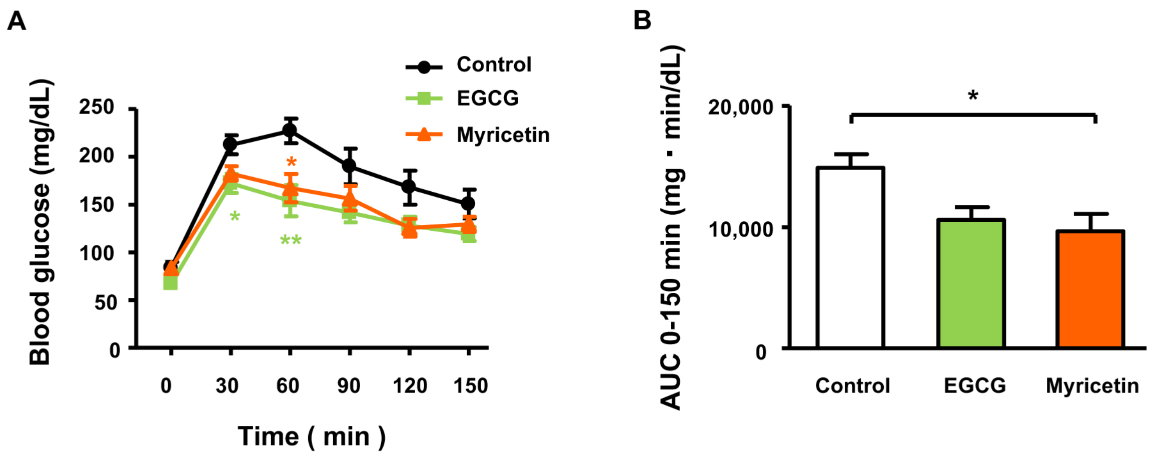

Fig. S5. Effect of EGCG or myricetin on postprandial blood glucose in mice. Mice were treated with EGCG or myricetin (500 mg/kg b.w.) in combination with soluble starch (2,000 mg/kg b.w.). (**A**) Postprandial blood glucose levels are shown at each time point after oral dosing of starch. (**B**) Calculated area under the curve (AUC) is shown during the whole time period (0–150 min). Turkey's Multiple Comparison Test, *$P < 0.05$, **$P < 0.01$ vs. Control.

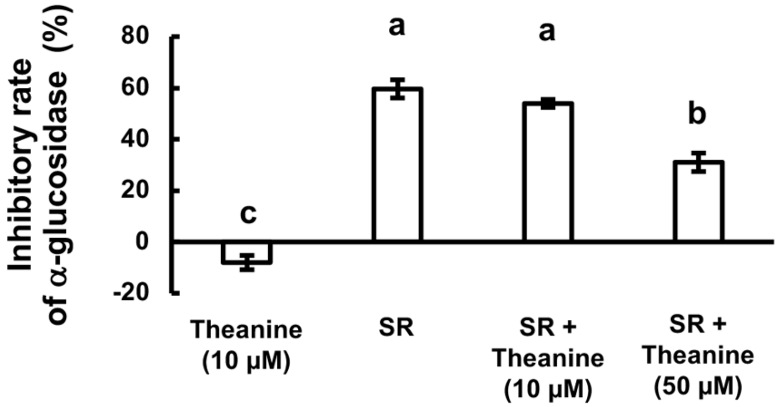

Fig. S6. Effect of theanine on α-glucosidase activity. The activity of α-glucosidase was measured using theanine or Sunrouge (SR) extract with or without theanine. Turkey's Multiple Comparison Test, n=3. Different letters are statistically different, $P < 0.05$.

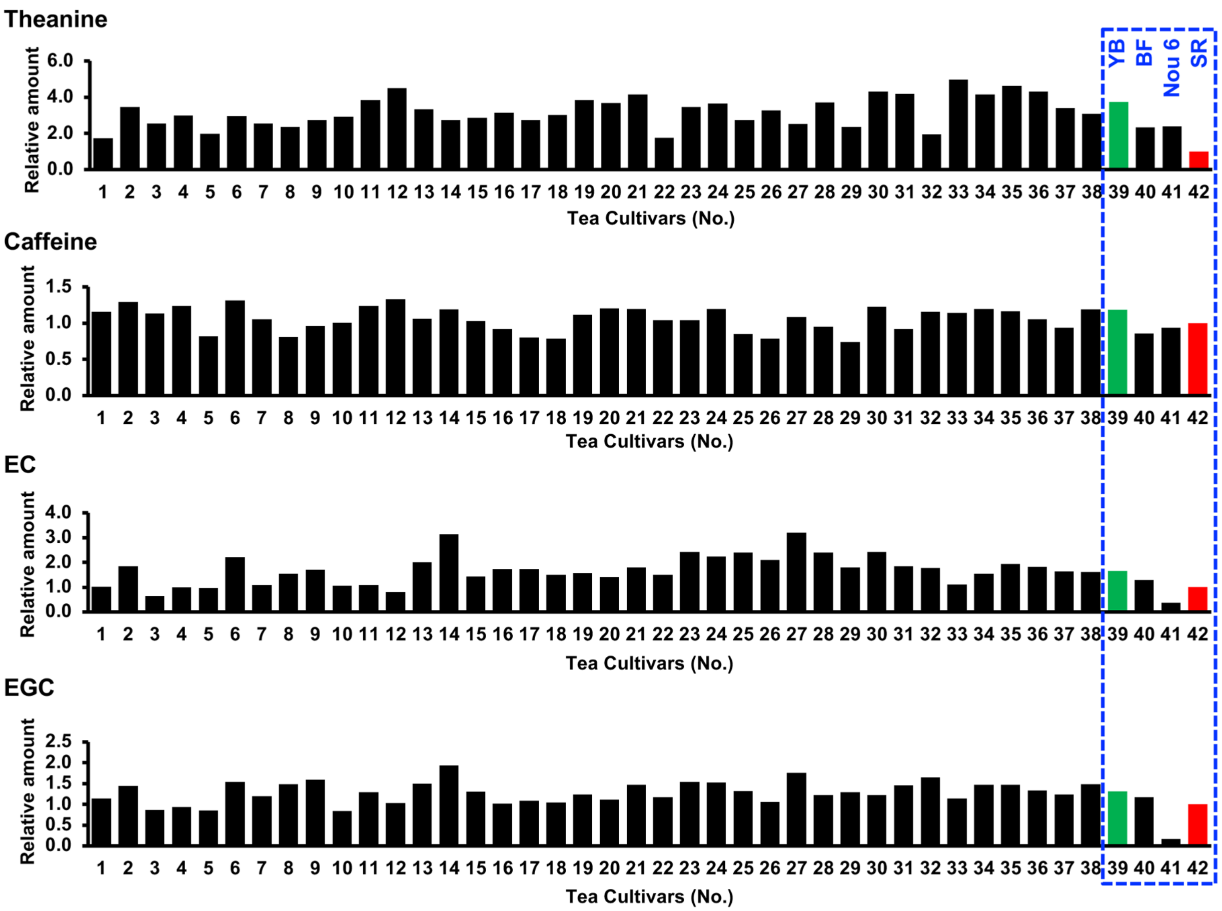

Fig. S7. The relative amount of the representative compounds showing negative correlation with α-glucosidase inhibitory rate in 42 green tea extracts. The relative amounts were calculated from the intensity data of MS profiles of 42 green tea cultivars (Supplementary Table S2) and represented as the relative value of each cultivar to the Sunrouge. SR: Sunrouge; Nou 6: Cha Chuukanbohon Nou 6; BF: Benifuuki; YB: Yabukita.
